# Multimodal Neuroimaging Approach to Variability of Functional Connectivity in Disorders of Consciousness: A PET/MRI Pilot Study

**DOI:** 10.3389/fneur.2018.00861

**Published:** 2018-10-18

**Authors:** Carlo Cavaliere, Sivayini Kandeepan, Marco Aiello, Demetrius Ribeiro de Paula, Rocco Marchitelli, Salvatore Fiorenza, Mario Orsini, Luigi Trojano, Orsola Masotta, Keith St. Lawrence, Vincenzo Loreto, Blaine Alexander Chronik, Emanuele Nicolai, Andrea Soddu, Anna Estraneo

**Affiliations:** ^1^IRCCS SDN, Istituto di Ricerca Diagnostica e Nucleare, Naples, Italy; ^2^Coma Science Group, GIGA-Research, University and University Hospital of Liege, Liege, Belgium; ^3^Department of Physics and Astronomy, Brain and Mind Institute, Western University, London, ON, Canada; ^4^Neurorehabilitation Unit and Research Laboratory for Disorder of Consciousness, Maugeri ICS, IRCCS, Telese Terme, Italy; ^5^Department of Psychology, University of Campania “Luigi Vanvitelli”, Caserta, Italy; ^6^Lawson Health Research Institute London, Medical Biophysics, University of Western Ontario, London, ON, Canada

**Keywords:** PET/MRI, unresponsive wakefulness syndrome, minimally conscious state, diagnosis, brain connectivity, resting-state fMRI, graph theory, glucose metabolism

## Abstract

Behavioral assessments could not suffice to provide accurate diagnostic information in individuals with disorders of consciousness (DoC). Multimodal neuroimaging markers have been developed to support clinical assessments of these patients. Here we present findings obtained by hybrid fludeoxyglucose (FDG-)PET/MR imaging in three severely brain-injured patients, one in an unresponsive wakefulness syndrome (UWS), one in a minimally conscious state (MCS), and one patient emerged from MCS (EMCS). Repeated behavioral assessment by means of Coma Recovery Scale-Revised and neurophysiological evaluation were performed in the two weeks before and after neuroimaging acquisition, to ascertain that clinical diagnosis was stable. The three patients underwent one imaging session, during which two resting-state fMRI (rs-fMRI) blocks were run with a temporal gap of about 30 min. rs-fMRI data were analyzed with a graph theory approach applied to nine independent networks. We also analyzed the benefits of concatenating the two acquisitions for each patient or to select for each network the graph strength map with a higher ratio of fitness. Finally, as for clinical assessment, we considered the best functional connectivity pattern for each network and correlated graph strength maps to FDG uptake. Functional connectivity analysis showed several differences between the two rs-fMRI acquisitions, affecting in a different way each network and with a different variability for the three patients, as assessed by ratio of fitness. Moreover, combined PET/fMRI analysis demonstrated a higher functional/metabolic correlation for patients in EMCS and MCS compared to UWS. In conclusion, we observed for the first time, through a test-retest approach, a variability in the appearance and temporal/spatial patterns of resting-state networks in severely brain-injured patients, proposing a new method to select the most informative connectivity pattern.

## Introduction

The improvements of medical interventions in the acute and post-acute phase of severe acquired brain injury and the failure of treatments to restore brain functions keep increasing the number of patients with prolonged disorders of consciousness (DoC) (1). These severe clinical conditions entail heavy ethical and social implications, impact health care policies and determine strong psychological distress in patients' families (2–4). Distinguishing patients in unresponsive wakefulness syndrome, UWS [i.e. patients showing eyes opening but no behavioral evidence of consciousness (5)] from patients in minimally conscious state, MCS [i.e., patients showing minimal, inconsistent but clearly discernible intentional behaviors (6)] is pivotal for decision making in the entire care pathway of patients with DoC. Indeed, patients in MCS are more likely to have a better outcome (7, 8) and a higher probability of clinical response to therapeutic interventions than patients in UWS (9–11). However, in spite of the evolution of neuroscientific and medical understanding on DoC, the clinical recognition of volitional behavior still remains a very difficult task (8, 12).

Patients' clinical signs of consciousness are frequently variable across days and even within the same day (13). These inconsistencies have been often linked to temporal fluctuations of vigilance/awareness. For this reason, at least five repeated behavioral assessments by means of validated assessment tools, such as Coma Recovery Scale-Revised (CRS-R) (14), are strongly recommended for improving diagnostic accuracy (15).

However, behavioral assessment might be complicated by possible co-existing severe visuo-perceptual, motor or language disabilities that limit clinical expression of consciousness (7, 16). In this context, a multimodal diagnostic approach, combining clinical and instrumental evaluations, could help detecting signs of consciousness and making a correct diagnosis (17–19). Neuroimaging methods, particularly those not requiring patients' active response, such as resting-state functional MRI (rs fMRI) or 18F FDG-PET, can recognize residual neural activity and functional connectivity into resting state networks (RSNs), such as the default-mode network (DMN), specifically associated with awareness level in such patients, independently from their abilities to produce overt purposeful behaviors (20–22). Moreover, multimodal imaging integration allows collecting a plethora of information undetectable at patients' bedside, but only simultaneous acquisition of neuroimaging data can assure inter-modality comparability of the findings extracted within the same temporal framework, thus reducing the influence of clinically fluctuations typical of patients with DoC. Additionally, the simultaneous acquisition of structural and functional data by hybrid imaging techniques like PET/MR can improve the patient's compliance, by shortening imaging sessions and reducing logistic issues (23).

The present clinical and neuroimaging pilot study aimed at: (1) investigating possible variability in brain functional connectivity in two distinct fMRI acquisitions within one neuroimaging exam through a test-retest approach; (2) evaluating the relationships of spontaneous functional brain activity with metabolic activity in different levels of consciousness.

For these purposes we combined simultaneous neuroimaging methods (fMRI and PET) and repeated rs-fMRI acquisition in a sample of three severely brain-injured patients with different level of consciousness in stabilized clinical diagnosis of UWS, MCS and emergence from MCS [EMCS, i.e., patient who recovered functional communication or/and functional object use; (5, 6)].

## Materials and methods

### Participants

We screened for the study severely brain-injured patients consecutively admitted to the neurorehabilitation Unit at Maugeri Clinical and Scientific Institutes, in Telese Terme (Italy) from February 2017 to July 2017, fulfilling the following inclusion criteria: (i) clinical diagnosis of UWS, MCS or EMCS according to standard diagnostic criteria (5, 6); (ii) time from onset longer than 1 month; (iii) traumatic, vascular or anoxic brain injury. We excluded from the study patients with: (i) severe pathologies independent from the brain injury (e.g., premorbid history of psychiatric or neurodegenerative diseases); (ii) mixed etiology (e.g., both traumatic and anoxic); (iii) not stabilized and severe general clinical conditions; (iv) contra-indication for MRI (e.g., ferromagnetic aneurysm clips, pacemaker); (v) large brain damage (>50% of total brain volume), as stated by a certified neuroradiologist, and motion parameters >3 mm in translation and 3° in rotation. Patients were also excluded if their clinical diagnosis had changed in the week before the neuroimaging acquisition.

The study was approved by the local Ethics Committee of IRCCS Pascale (Protocol number: 3/15), and performed according to the ethical standards laid down in the 1964 Helsinki Declaration and its later amendments. Written informed consent was obtained from the legal guardian of patient.

### Experimental procedures

#### Clinical assessment

One week before and one week after neuroimaging recording, all enrolled patients underwent at least five clinical evaluations, using the Italian version of the CRS-R (24), in order to confirm stabilized clinical diagnosis of UWS, MCS or EMCS and to gather the best CRS-R total score. Patients' consciousness level (measured by CRS-R total and sub-scores) was also assessed in the “neuroimaging” day by one skilled psychologist (OM) (Table S1).

#### Neurophysiological evaluation

Standard EEG and event related potentials (ERP) were recorded to complement behavioral assessment and to reduce risk of misdiagnosis. For this purpose we acquired neurophysiological exams at patients' bed in 2 days in the week before PET/MRI session and in 2 days in the week after neuroimaging exam, and the best organization of EEG background activity and reactivity was considered for classification of neurophysiological patterns, complementing patients' clinical diagnosis. In the presence of artifacts in more than 50% of EEG recording time, EEG acquisition was repeated in the day after. Two skilled clinical neurophysiologists (VL and SF, blinded to patients' etiology, clinical diagnosis and CRS-R score) reviewed neurophysiological exams.

*Standard EEG* was recorded by 19 electrodes placed on the scalp, according to international 10–20 system (O1, O2, Pz, P3, P4, T5, T6, C3, C4, Cz, T3, T4, Fz, F3, F4, F7, F8, Fp1, and Fp2). We recorded EEG for (at least) 35 min, according to standard procedure of eye-closed waking rest, with filter settings 0.53–70 Hz, and notch filter on. For the analysis of predominant activity, forced eye closing was obtained by cotton wool in awake patient (spontaneous eye opening). To analyse EEG reactivity, eye opening and (forced) eye closing were alternated three times during EEG recording. We classified EEG background activity on the basis of frequency and amplitude of predominant cortical activity present in >50% of recordings, into one of five severity categories, according to criteria recently proposed for patients with prolonged DoC [(25), Appendix 1].

*ERP* were obtained by means of a simple “oddball” paradigm using auditory stimulation and classified as “present” when P300 cortical response was recorded; in presence of N100 component the exam ERP was considered “absent,” whereas lack of N100 was considered as a not reliable exam (26).

#### PET/MRI acquisition protocol

PET/MRI data were simultaneously acquired in the resting state using a Biograph mMR tomograph (Siemens Healthcare, Erlangen, Germany) designed with a multi-ring LSO detector block embedded into a 3 T magnetic resonance scanner. Vacuumed pillows were used to minimize head movements within the scanner. The PET/MRI was acquired in the morning after customary nursing procedures. Moreover, we used some strategies to ensure patients' best vigilance state by: (i) stopping possible sedative drugs (such as benzodiazepine) 15 h before scanning; (ii) administering CRS-R vigilance protocol (14) before PET/MRI acquisition and during neuroimaging exam at the end of first resting state MRI acquisition; (iii) monitoring eyes opening by means of a video camera located into MRI scanner. In case of appearance of clinical signs of possible drowsiness (i.e., persistence of eye closing), MRI acquisition was stopped and CRS-R vigilance protocol was administered.

Nominal axial and transverse resolution of the PET system was 4.4 and 4.1 mm FWHM, respectively, at 1 cm from the isocenter. Additional technical details on the scanner are reported elsewhere (27).

A dynamic brain PET study was performed after the intravenous bolus administration of 18F-fluorodeoxyglucose (18F-FDG) tracer. PET and rs-fMRI data acquisition started simultaneously following the i.v.injection of 5 MBq/Kg of 18F-FDG.

No food or sugar were administered to the subjects for at least 6 h prior to FDG injection. Blood glucose was measured at arrival at the PET center in all cases, and FDG was injected only if glycaemia was below 120 mg/dl.

The PET data were acquired in list mode for 60 min; matrix size was 256 × 256. PET emission data were reconstructed with ordered subset-expectation maximization (OSEM) algorithm (21 subsets, 4 iterations) and post-filtered with a three-dimensional isotropic gaussian of 4 mm at FWHM. Attenuation correction was performed using MR-based attenuation maps derived from a dual echo (TE = 1.23–2.46 ms) Dixon-based sequence (repetition time 3.60 ms), allowing for reconstruction of fat-only, water-only and of fat–water images (28).

During PET acquisition, the following MRI sequences were sequentially run:

- First rs-fMRI acquisition (named “T1”) by a T2^*^-weighted single-shot EPI sequence (voxel-size 4 × 4 × 4 mm3, TR/TE = 1000/21.4 ms, flip angle = 82°, 480 time points, FOV read = 256 mm, multiband factor = 2, distance factor = 0, TA = 8′06″);
- Three-dimensional T1-weighted magnetization-prepared rapid acquisition gradient-echo sequence (MPRAGE, 240 sagittal planes, 256 × 214 mm field of view, voxel size 0.8 × 0.8 × 0.8 mm3, TR/TE/TI 2400/2.25/1000 ms, flip angle 8°, TA = 6′18″);
- Three-dimensional T2-weighted sequence (240 sagittal planes, 256 × 214 mm field of view, voxel size 0.8 × 0.8 × 0.8 mm3, TR/TE 3370/563ms, TA = 6′46″);
- Three-dimensional fluid attenuation inversion recovery (FLAIR, 160 sagittal planes, 192 × 192 mm field of view, voxel size 1 × 1 × 1 mm3, TR/TE/TI 5000/334/1800 ms, TA = 6′42″);
- Second rs-fMRI acquisition (named “T2”) by a T2^*^-weighted single-shot EPI sequence (voxel-size 4 × 4 × 4 mm3, TR/TE = 1000/21.4 ms, flip angle = 82°, 480 time points, FOV read = 256 mm, multiband factor = 2, distance factor = 0, TA = 8′06″).

In addition, during the same scanning session, axial diffusion weighted images were also acquired for clinical purpose. The two rs-fMRI acquisitions (T1 and T2) were separated by a 30 min interval.

#### fMRI and FDG-PET processing

Resting state fMRI analysis was performed based on a methodology fully described by Ribeiro and colleagues (29). Independent component analysis (ICA) (30) followed by template matching to identify RSNs and machine learning classification to automatically recognize a neuronal source was used. We extracted the weighted graphs for each of the nine networks of interest as described in the paper (29) and calculated the graph strength (GS) for each of the 1015 nodes. Finally, for each network we calculated the correlation between the GS and the metabolic values.

Nine RSNs of interest are recognized: auditory, default mode network (DMN), extrinsic-control network left (ECNL), extrinsic-control network right (ECNR), salience, sensorimotor, visual lateral (VL), visual medial (VM) and visual occipital (VO). The RSNs are assigned as the components with maxima goodness-of-fit (similarity test) when compared to a binary predefined template while considering all the RSNs simultaneously. The templates for each RSN were selected by an expert after visual inspection from a set of spatial maps resulting from a Group ICA decomposition performed on 12 independently assessed controls and were confirmed by another expert for accuracy of structural labeling (31). Subsequently a classifier trained on an 11-dimensional space called “fingerprint,” that provides both spatial (i.e., degree of clustering, skewness, kurtosis, spatial entropy) and temporal information (i.e., one-lag autocorrelation, temporal entropy, power of five frequency bands: 0–008 Hz, 0.008–0.02 Hz, 0.02–0.05 Hz, 0.05–0.1 Hz, and 0.1–0.25 Hz) of the ICs, is used to select only the neuronal components from the extracted networks (31). Signals arising from changes in local hemodynamics which result solely from alterations in neuronal activity represented by low-frequency (0.01–0.05 Hz) are called neuronal signals. Non-neuronal signals for fMRI data represents cardiovascular signal dominated by higher frequency and head movement.

Once the neuronal components are identified, a graph theoretical approach was applied on the ICs (GraphICAr, BraiNet-Brain Imaging Solution Inc.-Sarnia, ON, Canada) to visualize and calculate the graph properties of each network (30, 32, 33). GraphICAr is a software in which single-subject ICA with 30 components was ordered using the infomax algorithm as implemented in the Group-ICA of fMRI toolbox (https://scicrunch.org/resolver/RRID:SCR-001953RRID: SCR-001953; http://mialab.mrn.org/software/gift/). Instead of working at the voxel level (around 100,000 voxels) for the analysis, the cortex was parcellated into 1015 regions of interests (ROIs) with anatomical meaning, using the Lausanne 2008 Atlas with functions from the Connectome Mapping Toolkit (34). Each ROI is considered as a node of a graph; the edges connecting the nodes typically carry weights describing the correlation, or the degree of connectivity between each pair of nodes. After decomposing the whole brain to components using ICA, the weighted matrices (w_ij_) for each of the nine components are obtained by calculating the edge weights using the Equation (1):

(1) $$\begin{array}{l} w_{ij}=\left|z_{i}\right|+\left|z_{j}\right|-\left|z_{i}-z_{j}\right| \end{array}$$

where *w*_ij_ represents the edge weight between nodes “*i*” and “j,” and *z*_*i*_*, z*_*j*_ are the *z*-values which are obtained from the scalar map of the independent component of interest for the nodes “*i*” and “j,” respectively.

Furthermore, the two fMRI acquisitions which were obtained for all three patients within a time interval of 30 min and the FDG-PET data, were manually co-registered with their structural images. These data, along with the concatenated data (combined T1 and T2), underwent an automated pipeline in GraphICAr, which includes further minute realignment and adjustment for movement-related effects, fine co-registration, segmentation of the structural and FDG-PET image, and spatial normalization into standard stereotactic Montreal Neurological Institute (MNI) space as performed in SPM8. Considering the relevance of motion for these dataset, as already reported in Soddu et al. (32), motion parameters such as the mean displacement (Δ) and the displacement speed (Σ) during the full acquisition were calculated using the equations explicitly given by Equations 2, and 3,

(2) $$\begin{array}{l} \Delta =\;\langle \sqrt{TraX^{2}+TraY^{2}+TraZ^{2}+RotX^{2}+RotY^{2}+RotZ^{2}}\rangle \end{array}$$

(3) $$\begin{array}{l} \Sigma =\langle \sqrt{\Delta_{TR}TraX^{2}+\Delta_{TR}TraY^{2}+\Delta_{TR}TraZ^{2}+\Delta_{TR}RotX^{2}+\Delta_{TR}RotY^{2}+\Delta_{TR}RotZ^{2}}\rangle \end{array}$$

Where Δ_*TR*_ represents the variation of a parameter over a TR.

Motion curves were regressed out from the fMRI data when performing the preprocessing using Art repair (https://scicrunch.org/resolver/RRID:SCR-005990RRID:SCR-005990; http://cibsr.stanford.edu/tools/human-brain-project/artrepair-software.html), but not the motion parameters. Instead these parameters were just calculated to estimate how much the patients have moved in the scanner during each acquisition.

Segmentation of the images in GraphICAr was performed at the subject level to create its own segmentation (35). Following these preprocessing steps, ICA was applied and *w*_*ij*_ matrices for each of the nine networks were obtained. Simultaneously the scalar maps of the FDG-PET for the 1015 parcellated regions of the cortex were obtained.

The *w*_ij_ matrices which have the dimensions of 1015 × 1015 were thresholded such that the *w*_ij_ values that are less than the threshold were set to zero while the values greater than the threshold were kept as it is. Thresholds were selected from 0 to 1 in steps of 0.01 and the mean over the thresholded *w*_ij_ matrices were obtained. The graph strengths (*S*_*i*_) for each of the 1015 regions for all three subjects and for the nine networks were calculated from the thresholded *w*_ij_, using the Equation (4):

(4) $$\begin{array}{l} S_{i}=\sum_{j=1}^{N}W_{ij} \end{array}$$

where “N” is the total number of regions.

Graph strength (GS) was tested at the network level for proportionality with metabolic activity. In particular, only regions with GS values greater than the thresholded GS (values greater than half of the maximum GS value for the network of interest) were visualized and selected for subsequent calculations.

Non-neuronal networks were removed and the networks classified as neuronal were chosen for the analysis. Using the GS values, the regions belonging to each network (mask), regions outside the network and regions missing in the network for patients in EMCS, MCS and UWS were plotted in different colors for T1, T2 and concatenated data. In the case where the networks from both acquisitions were neuronal, the ratio of fit (ROF) (Equation 5), a measure assessing accuracy of network representation in the analysis, was calculated (Table 1).

(5) $$\begin{array}{l} ROF=\;\frac{\left(regions\;inside\;the\;mask-regions\;outside\;the\;mask\right)}{\left(total\;number\;of\;regionswhich\;should\;belong\;to\;the\;mask\right)} \end{array}$$

**Table 1:** ROF values calculated from regions belonging to the GS values, separated into the regions belonging to the network itself and outside the network.

**Networks**	**EMCS**							**MCS**							**UWS**						
	**T1**			**T2**				**T1**			**T2**				**T1**			**T2**			
	**In**	**Out**	**ROF**	**In**	**Out**	**ROF**	**ΔROF**	**In**	**Out**	**ROF**	**In**	**Out**	**ROF**	**ΔROF**	**In**	**Out**	**ROF**	**In**	**Out**	**ROF**	**ΔROF**
Auditory				85	87	−0.01	0.01	41	26	0.06	36	9	0.10	−0.04				108	162	−0.20	0.20
DMN	81	13	0.19	78	76	0.01	0.18	82	8	0.21	107	44	0.18	0.03	53	123	−0.20				−0.20
ECNL	46	27	0.12	57	70	−0.08	0.20	20	142	−0.80	48	88	−0.26	−0.54	29	158	−0.84				−0.84
ECNR				11	0	0.08	−0.08	30	49	−0.15	46	125	−0.60	0.45							
Salience				30	108	−0.67	0.67	58	303	−2.11	46	228	−1.57	−0.54	16	52	−0.31	37	235	−1.71	1.40
Sensorimotor	39	213	−1.71	2	27	−0.25	−1.46	42	30	0.12	41	13	0.27	−0.15	36	69	−0.32				−0.32
VL				35	179			4	79	−0.56	10	75	−0.49	−0.07				42	108	−0.50	0.50
VM	125	37	0.32	37	22	0.05	0.27	93	21	0.26	48	53	−0.02	0.28							
VO								41	54	−0.07	93	57	0.20	−0.27				6	14	−0.04	0.04

“*In” and “Out” represent the total number of regions belonging to and outside of the network. Values of the non-neuronal networks are not presented*.

Positive value of ROF indicates a high resemblance of the network (higher the value, better the resemblance), while a negative value means a distorted network. The difference between ROF (ΔROF) values for T1 and T2 acquisitions of each RSN was used to assess IC variability.

Scalar maps representing the GS for each network were presented by choosing the acquisition with the highest ROF value (best finding) between the two acquisitions and was used for further analysis.

As recalled above, DMN includes several cortical regions whose metabolic activity is thought to be related to level of consciousness (20–22). We believe that presenting the GS directly on the normalized structural images, especially for the DMN has relevance, because it shows the anatomical pattern of the network and permits to visualize the level of disruption or completeness. However, we believe it would be too redundant to present the GS for all networks in the same modality. To ascertain whether the concatenated data or the data corresponding to the acquisition with the best network between T1 and T2 provided the best representation of the network, both concatenated and best acquisition data were plotted.

### Statistical analysis

Correlation between FDG-PET and GS was performed to measure the similarity between the FDG-PET metabolic maps and the GS activity maps for the whole brain. In order to get the most representative value of the GS for each region from all the networks, the maximum value out of all the neuronal networks for that region was chosen. The z-scores of the GS and PET for each region were calculated and the scatter plots of FDG-PET versus GS were presented for the best and concatenated data for the three patients. “Corrcoeff” function as implemented in MATLAB, which returns the Pearson correlation value (r) between the FDG-PET and GS of the 1015 parcellated ROI was calculated and presented along with the statistical p value for testing the null-hypothesis of no correlation. The *p*-value is computed by transforming the correlation into a t-statistical variable having N−2 degrees of freedom, with N the number of data points. Furthermore, the distribution of the GS for the best and concatenated data and FDG-PET were estimated.

## Results

### Clinical features

**Figure 1:**
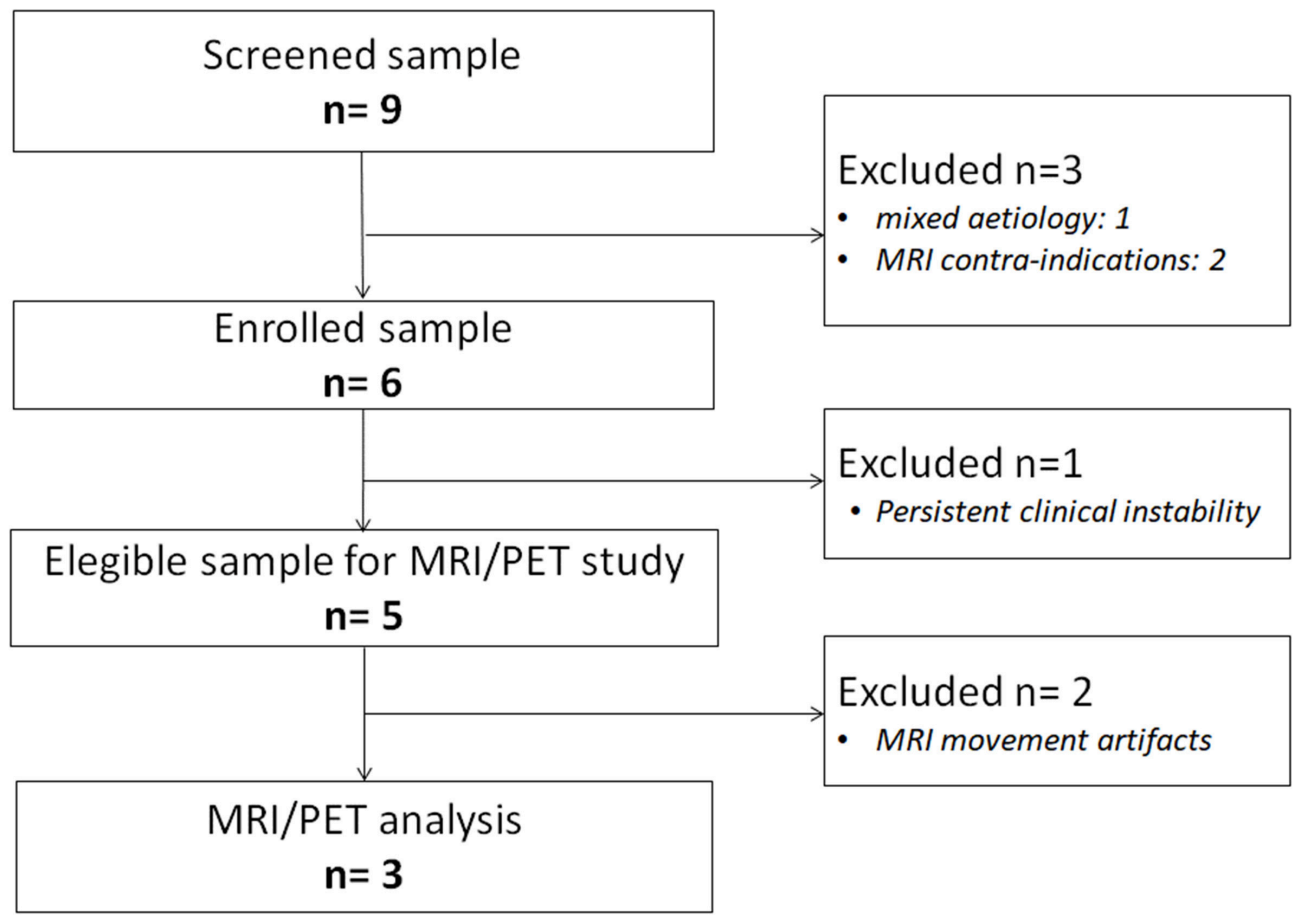
Flow chart of patient selection in each step of the study.

**Figure 2:**
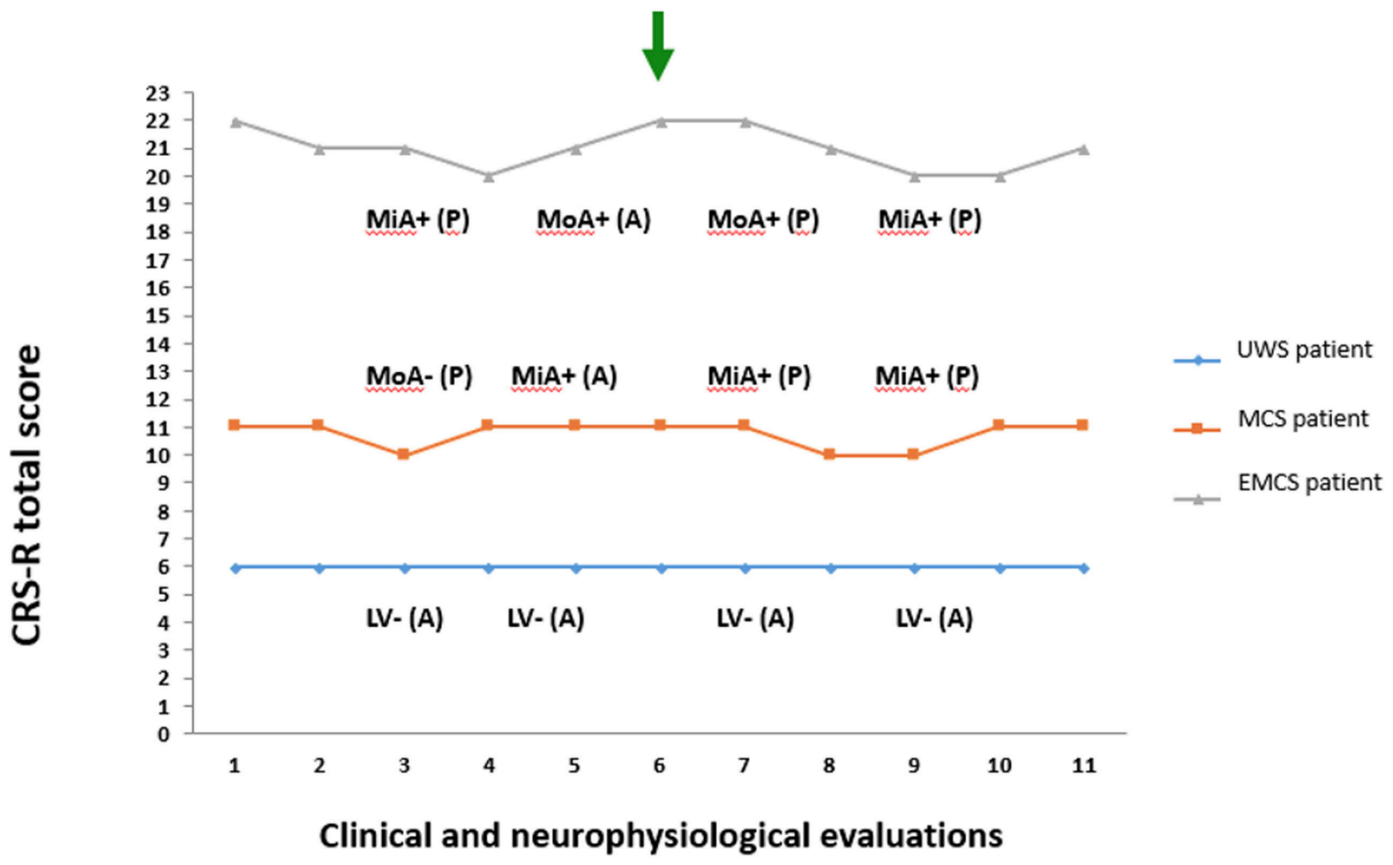
Coma Recovery Scale-Revised total score and neurophysiological (EEG and evoked related potential) evaluations recorded in the 3rd and 5th day before PET/fMRI exam and in the 7th and 9th day after the PET/fMRI exam. The green arrow marks the day of neuroimaging acquisition. The blue diamond and line denote the patient in unresponsive wakefulness syndrome (UWS). The orange square and line denote the patient in minimally conscious state (MCS). The gray triangle and line denote the patient emerged from MCS (EMCS). CRS-R, Coma Recovery Scale-Revised; P, presence of P300 on evoked related potential; A, absence of P300 on evoked related potential; +, presence of EEG reactivity to eye opening and closing; –, absence of EEG reactivity to eye opening and closing; MiA, mildly abnormal EEG background activity; MoA, moderately abnormal EEG background activity; DS, Diffuse slowing EEG background activity; LV, Low voltage EEG background activity.

From a sample of nine severely brain-injured patients, we could consider for PET/fMRI analysis two representative patients with prolonged DoC and one patient emerged from MCS (Figure 1). Detailed descriptions of patients' clinical features are provided in Appendix and the CRS-R total and subscores in Supplemental Material (Table S1). In synthesis, one anoxic patient was in UWS (F, 43 year old; time since injury: 8 months; best CRS-R total score: 6; CRS-R total score at neuroimaging study: 6), 1 traumatic patient in MCS (M, 18 year old; time since injury: 3 months; best CRS-R total score: 11; CRS-R total score at neuroimaging study: 11), and 1 anoxic patient emerged from MCS 25 days before the neuroimaging study (M, 57 year old; time since injury: 10 months; best CRS-R total score: 22; CRS-R total score at neuroimaging study: 22). The best CRS-R total scores collected in each patient in the weeks before and after the neuroimaging session and in the PET/MRI day are described in Figure 2.

### Neurophysiological findings

The best neurophysiological findings out of 4 EEGs and 4 ERPs recorded in each patient are summarized in Figure 2. In the patient in UWS we observed a poor organization of cortical activity with predominant EEG delta activity with amplitude less than 20 μV over most brain regions, not reactive to eye closing (i.e., Low Voltage, LV category) and lack of P300. In the patient in MCS we observed predominant reactive posterior theta EEG activity (amplitude >20 μV), with frequent posterior alpha rhythm (i.e., mildly abnormal, MiA category) in 3 out of 4 EEG recordings. A P300 cortical response was recorded at least following “oddball” paradigm in 3 out of 4 exams. In the patient in EMCS, a predominant reactive posterior theta EEG activity (amplitude ≥20 μV), with frequent posterior alpha rhythms (i.e., mildly abnormal, MiA EEG category) was recorded in 2 out of 4 EEG recordings. In all EEG acquisitions, the background activity showed reactivity to eye opening and closing. The “oddball” paradigm evoked a positive cortical component (i.e., P300) in 3 out of 4 exams.

### Within-session fMRI variability

**Figure 3:**
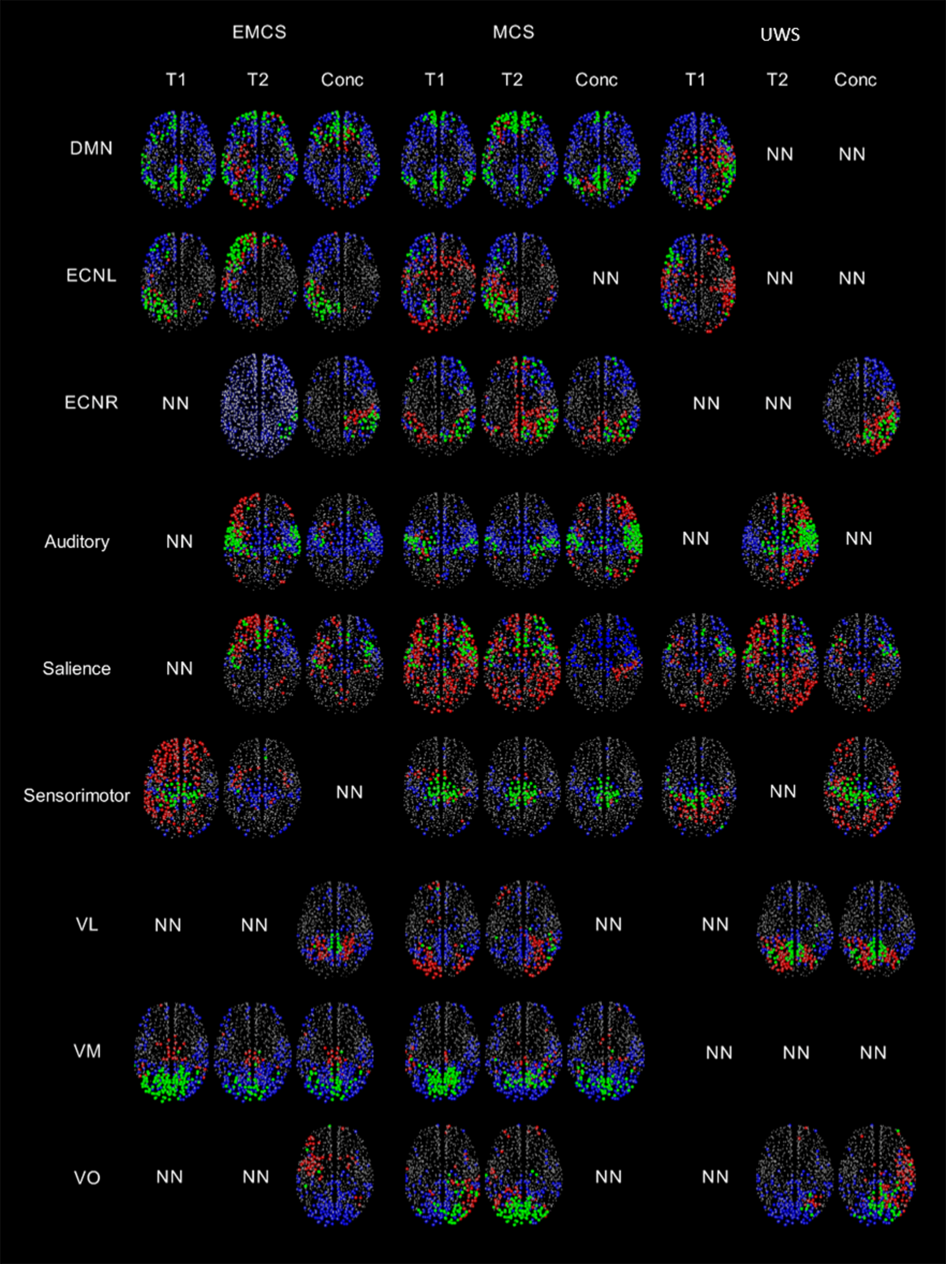
A visual representation of the regions highlighted by the thresholded GS (values greater than half of the maximum GS value for the network), separated by the regions within and outside the network for patients in EMCS, MCS and UWS for nine RSNs. Regions belonging to the network and having GS values greater than the thresholded GS are represented by green, regions which should be in the network but do not have GS values greater than the thresholded GS are represented by blue, regions outside the network but have GS values greater than the thresholded GS are represented by red color. NN represents non-neuronal networks. Here the size of the circle doesn't represent the value of the GS, all the regions with a GS value are plotted evenly.

In the patient in EMCS, the DMN appeared spatially preserved during the first (T1) rs-fMRI acquisition (ROF = 0.19 vs. ROF = 0.01 at T2), with a main neuronal component (Figure 3, Table 1). The ECN was well preserved in both acquisitions on the left, although in the T2 rs-fMRI there was some superposition due to other regions, as shown by the negative value of ROF (−0.08 vs. ROF = 0.12 atT1), while it appeared inconsistent on the right, and not neuronal in T1 acquisition. Auditory and salience networks were partially preserved and evident only in the T2 scan. Moreover, the auditory appeared more lateralized to the left (Figure 3, Table 1). Sensorimotor was spatially preserved in T1, where it appeared wider for the co-activation of many nodes outside the network (ROF = −1.71). VL and VO were recognized as not neuronal in both scans, while VM appeared well preserved with a better spatial pattern in T1 acquisition (ROF = 0.32 vs. 0.05 at T2) (Figure 3, Table 1).

In the patient in MCS, the preservation of DMN was clear in both acquisitions (T1 and T2), with ROF values of 0.21 and 0.18, respectively. The ECN was partially recognized for both hemispheres in both acquisitions although the number of nodes outside the network was high as highlighted by the negative values of ROF (Figure 3, Table 1). Auditory and sensorimotor networks appeared well preserved in both acquisitions, with a complementary mirrored visualization for the auditory one between T1 and T2. On the other hand, the salience network was evident in both acquisitions, but with a spread co-activation of nodes outside the network (ROF = −2.11 and −1.57 at T1 and T2, respectively) (Figure 3, Table 1). As for the three visual networks, while the VL was recognized as neuronal in both T1 and T2, but with a poor spatial representation, both VM and VO appeared temporal and spatially preserved with a better visualization of VM at T1 (ROF = 0.26 vs. −0.02 at T2), and of VO at T2 (ROF = 0.20 vs. −0.07 at T1) (Figure 3, Table 1).

In the patient in UWS, ECNR was not found in both acquisitions, while the DMN and ECNL were partially detected in T1, although with high number of regions outside the networks revealed by negative values of ROF (−0.20 and −0.84 respectively) (Figure 3, Table 1). In the same manner, the salience network was detected in both acquisitions along with more regions outside the network (ROF = −0.31 and −1.71 respectively). Auditory and sensorimotor networks were identified only in one acquisition, with a higher number of regions belonging outside of the network (ROF = −0.20, −0.32) (Figure 3, Table 1). Finally, out of the three visual networks, contrary to the other two patients, VM was not identified in either acquisition. VL and few regions of VO were detected in the second acquisition (Figure 3, Table 1).

Summarizing, a wider variability was found for ICs representation in the patient in EMCS (mean |ΔROF| = 0.32) and in UWS (mean |ΔROF| = 0.39) than in MCS case (mean |ΔROF| = 0.26).

### Mutual fMRI findings

When considering the best finding between the two rs-fMRI acquisitions (T1 and T2) for each network, ICA components classified as neuronal networks were 61, 100, and 44% for patients in EMCS, MCS, and UWS (Figure S1), respectively. In the patient in EMCS, the DMN and VM networks were fully preserved, and most regions of ECN and sensorimotor were detected as well (Figure S1). Regions belonging to the spatial pattern and extra regions were identified in the auditory network, while mainly regions that did not belong to the salience network were detected. Out of the three visuals, only the VM was identified as neuronal with a good spatial representation of the network. In the patient in MCS (Figure S1), almost all the networks except the salience network seem to be well preserved, despite ECNR being spread out to both hemispheres and VL being lateralized. In the patient in UWS, the spatial patterns of most of the networks (except the sensorimotor and VM) were not well defined (Figure S1).

The head displacement of the patient in EMCS in the scanner during both T1 and T2 acquisitions was 0.09, whereas for the patient in MCS they were 0.03 and 0.06, respectively. Overall the lowest displacement was observed for the patient in UWS with the values of 0.02 and 0.04 respectively (Figure S2). The speed of the patients in the scanner for the T1 and T2 acquisitions of patient in EMCS were 2.0 × 10^−4^ and 3.7 × 10^−4^, for patient in MCS: 2.9 × 10^−5^ and 1.9 × 10^−4^, and for patient in UWS: 6.3 × 10^−4^ and 5.6 × 10^−5^ respectively (Figure S2).

Looking at the spatial distribution of the three most representative axial slices of the GS implemented on the normalized structure of the DMN network, in the best acquisition of both EMCS and MCS, this network was preserved throughout the brain, while in the patient in UWS, the GS seems to be highlighted mostly in the areas outside the network (Figure S3). In the concatenated case, the network was present only in the patient in MCS, but in the patient in EMCS, only the frontal part was found while in the patient in UWS, the network was not even recognized. In this figure, the GS values from 0.5 to 1 were represented in the jet color notation.

### Functional-metabolic correlation

Considering the functional-metabolic correlation in these patients, a significant positive correlation (*p* < 0.05) existed between the FDG-PET and GS for all three patients when considering the whole brain (Figure S4). In the best ICs pattern, EMCS had the highest correlation (*r* = 0.19, *p* < 0.01), whereas in the concatenated case, the MCS had the highest correlation (*r* = 0.21, *p* < 0.01). This implies that, when both results are reasonably good, concatenated data seems to give a better representation. It's evident that overall the patient in UWS had the lowest correlation out of all three patients with correlation values of 0.08 (*p* = 0.02) and 0.10 (*p* < 0.01) for the best and concatenated data respectively. The positive skewness value of 0.31 for the FDG-PET distribution (Figure S5) of the patient in EMCS indicated that there were many regions metabolically more active than the mean PET value. In the UWS instance, many regions were lower or similar in activity to the mean value, as confirmed by the negative skewness value of −0.01.

## Discussion

In the present pilot study, we investigated variability within a period of about 30 min in brain functional connectivity in three severely brain-injured patients (two patients still with DoC and one patient emerged from DoC). Moreover, we employed a methodological approach based on the graph theory and independent component analysis, to decompose brain connectivity maps in different networks and to correlate it to glucose metabolic activity simultaneously acquired through a PET/MRI scanner. We could demonstrate several differences between the two rs-fMRI acquisitions affecting in a different way each network and with a different variability in the three patients.

Functional connectivity assessed among the nodes belonging to different resting-state networks is sensitive to normal aging (36) and levels of consciousness (37–40), representing a potential biomarker of disease in longitudinal studies (41). Although being quite variable during pathological conditions, RSNs examined with a test-retest approach are thought to be highly reproducible within the same sample (42, 43). In a recent paper (44), co-activation patterns approach has been used in DoC patients, demonstrating heterogeneous spatial reconfiguration of DMN but also similar fluctuations of the BOLD signal in patients compared to control individuals. While these authors referred to BOLD signal oscillations during a single resting-state fMRI session, we scheduled two resting-state acquisitions with a 30-min interval, to investigate through a test-retest approach possible variability in functional connectivity within RSNs. Several differences were found between T1 and T2 session, with higher variability for the EMCS and the UWS case, compared to the patient in MCS. These findings apparently did not fit the substantial stability in the clinical diagnosis demonstrated by repeated behavioral assessments in the present brain-injured patients. However, we could speculate that this novel methodological approach is suitable to detect minimal fluctuations in brain connectivity not sufficient to determine relevant behavioral changes (i.e., by changes in clinical diagnosis), but nonetheless likely related to the variations detected by multiple CRS-R total scores and neurophysiological assessments. However, the nature and clinical significance of the fluctuations of the functional connectivity observed here remain to be established. Furthermore, multimodal investigations, possibly combining neuroimaging and neurophysiological assessment, are necessary to ascertain if variability in brain connectivity is associated to temporal variability of EEG activity characterizing patients with high probability of vigilance fluctuations (45).

On the basis of these considerations, we suggest that this innovative approach for neuroimaging analysis could permit clinicians to better identify the best functional brain performance, needed for the diagnostic classification of patients with high likelihood of clinical misdiagnosis. These findings could be extremely interesting, mainly for patients who are clinically diagnosed as UWS, where possible minimal and inconsistent signs of consciousness may not be recognized by behavioral assessments, leading to possible misdiagnosis (12, 46–48), and for detecting subtle signs of recovery of consciousness (8, 49, 50).

The same methodology should be applied to larger patient samples, also including a high number of patients without fluctuations of CRS-R total score, to comprehend which variations of functional connectivity might be related to substantial clinical fluctuations or to a basic variability of neuronal network.

The differences in spatial patterns observed in the two acquisitions within the same patient might be due to motion and artifacts. These artifacts affect the nine networks in different manner (32). However, the present findings suggest that not necessarily one acquisition is capable of detecting spared or impaired networks reliably. This observation suggests acquiring more than one acquisition during the scanning interval and to develop a gold standard for choosing the best one.

The GS scalar maps of most networks were more similar to the standard template of the networks in the patients in EMCS and MCS than in the patient in UWS. Specifically, all the networks of the patient in MCS and the important networks (but VL and VO) of the patient in EMCS were recognized. This implies that the brain functional organization was relatively preserved for the patients in EMCS and MCS. However, the auditory and salience networks had higher GS in regions outside the network likely in relation to the brain lesion. In the patient in MCS, although the salience network behaved as neuronal, the spatial pattern was not well-defined, suggesting that this network was distorted and metabolically impaired. Although seven out of the nine networks could be recognized in the patient in UWS, they had hyper-connectivity (confirmed by the negative ROF values), resembling non-normal condition. This might be related to the severe pathological condition of the patient in UWS affecting the spatial patterns of most networks (32).

A significant positive correlation was observed between the FDG-PET and GS for all three patients, although the r values were small. Overall, a higher correlation was observed for the patient in EMCS and MCS compared to the UWS case while using the concatenated data. The negative skewness value for the FDG-PET of the patient in UWS (FDG-PET values region by region were normalized by the global signal or mean all over the 1015 regions), is explained by the fact that there are only few regions with metabolic activity above the mean value. In the patient in EMCS, the distribution of the FDG-PET is tailed toward the left with a positive skewness value showing that there are several regions more metabolically active than the average, favoring conscious behavior.

### Limitations of the study

The present study had several limitations. First, we acknowledge that the low number of patients was a major limitation. We selected three patients with different clinical diagnosis (i.e., UWS, MCS and EMCS), to preliminarily investigate possible variability in fMRI connectivity in patients with different level of consciousness. The small sample size did not allow any generalization, but we hope that our preliminary study could serve as a starting point for devising multicenter studies on larger samples, comparing data of patients with different levels of consciousness, different etiologies and in different disease phases. Second, we could not calculate rigorous associations between patients' behavioral profiles (measured by repeated CRS-R assessments) and their possible brain connectivity variability, since the two features could not be measured in the same time window. Also, we did not perform clinical assessments immediately before and at the end of MRI acquisition since it could not ensure a strictly closed evaluation of possible patients' fluctuation in the two fMRI acquisitions.

May be the best tool to quantitatively assess even sub-clinical variations of cortical activity that could be correlated with repeated resting state fMRI seems to be prolonged EEG monitoring (45). However, we would underline that we enrolled patients in stabilized clinical diagnosis (even though in slightly fluctuated CRS-R scores), as demonstrated by repeated clinical assessments in the weeks before and after neuroimaging day, and with time from brain injury more than 1 month in order to minimize possible biases related to spontaneous clinical changes in the two different resting MRI acquisitions. Third, a lack of specific alertness level monitoring (such as EEG recording) during scanning acquisition could be a limit for the analysis within and between subjects, since we could not exclude variations in wakefulness as confounders for intrinsic functional connectivity analysis (51). However, we used some strategies to ensure patients' best vigilance state as described above.

Finally, the lack of a control group was a limitation of the present study, although the choice of the best reference group for patients with DoC is still debated (healthy subjects vs. injured patients that recovered consciousness, like for EMCS). Nevertheless, rs-fMRI functional connectivity metrics, mainly extracted by ICA, have demonstrated a high test-retest reproducibility (42). Moreover, other studies have demonstrated the potential of rs-fMRI functional-metabolic correlation assessed by simultaneous PET/MRI in healthy subjects (52), and in other neurological conditions, like Alzheimer disease (53).

## Conclusions

Since repeated acquisitions within 30 min showed relevant variability through a test-retest fMRI approach, we suggest performing multiple acquisitions within the same session to pick the best findings and possibly to compare these findings in longitudinal acquisitions. This procedure, together with the combined simultaneous acquisition of fMRI and PET, could provide useful information for improving characterization of patients with DoC. In a not well-defined number of patients with clinical diagnosis of unresponsive wakefulness syndrome, paraclinical testing (such as fMRI by active task or acquisition in resting state) could reveal cortically mediated cognitive functions (the so-called covert cognition). In this context our approach (i.e., double resting fMRI acquisitions combined with PET scanner) could help clinicians to increase the probability of detecting (spared) functional connectivity, which might provide diagnostic and prognostic information.

## Ethics statement

All procedures performed in the studies involving human participants were in accordance with the ethical standards of the institutional review boards of PLA Army General Hospital and with the 1964 Helsinki Declaration and its later amendments or comparable ethical standards.

## Author contributions

CC and SK: Study concept and design, analysis and interpretation of data, manuscript preparation; MA, OM, and EN: Imaging protocol design and acquisition; RM: Interpretation of data and manuscript revision; DR: Processing the data; LT: Revision of manuscript; VL: Acquisition of data, analysis and interpretation of data; SF: Neurophysiological data acquisition, analysis, and interpretation of data; MO: Clinical assessment, analysis and interpretation of data; BC and KS: Study concept and design; AS: Study concept and design, interpretation of data and critical revision of manuscript; AE: Study concept and design, interpretation of data and critical revision of manuscript for intellectual content.

### Conflict of interest statement

The authors declare that the research was conducted in the absence of any commercial or financial relationships that could be construed as a potential conflict of interest. The reviewer AD declared a shared affiliation, with no collaboration, with one of the authors CC to the handling Editor.

## Supplementary material

Figure S1 GS scalar maps of the nine RSNs of patients in EMCS, MCS, and UWS. From the two acquisitions, only the networks classified as neuronal are shown. When both acquisitions had neuronal components, the highest ROF value was used to choose the best spatial pattern of the network. The size of the circle represents the strength of the GS. The darker the circle, the higher the GS. Only the GS values greater than 0.5 of the maximum GS value of that network are plotted.

Figure S2 Motion curves illustrate translation (in mm) for *x* (blue), *y* (red), and *z* (orange) and rotation (in °) for pitch (blue), roll (red), and yaw (orange) parameters, and the time courses of each the nine RSNs (auditory, DMN, ECNL, ECNR, salience, sensorimotor, VL, VM, and VO) over 480 s.

Figure S3 Three most representative axial slices of the GS implemented on the normalized structure of the DMN network are presented for the three patients for the best functional pattern and concatenated data.

Figure S4 Scatter plots for the EMCS, MCS and patients in UWS showing the correlation between the FDG-PET and GS of the 1015 parcellated ROI. Solid line indicates the best linear fit to the data and on the northeast corner of each scatter plot the linear correlation value is reported along with its statistical *p*-value.

Figure S5 Distribution plots of GS for the best acquisition, concatenated data and FDG-PET for patients in EMCS, MCS and UWS.

Table S1 Coma Recovery Scale-Revised total and subscores in the three patients collected in the day of neuroimaging and 5 days in 1 week before and after.

Appendix 1 Classification criteria for visual analysis of EEG background activity.

## Abbreviations

DoC: disorder of consciousness
UWS: unresponsive wakefulness syndrome
MCS: minimally conscious state
CRS-R: Coma Recovery Scale-Revised
PET: positron emission tomography
FDG: fludeoxyglucose
fMRI: functional magnetic resonance imaging.
