# Supplementary figures and images for: Multimodal Neuroimaging Approach to Variability of Functional Connectivity in Disorders of Consciousness: A PET/MRI Pilot Study
Source: Front Neurol. 2018 Oct 18;9:861. doi: 10.3389/fneur.2018.00861 (PMC6200912; doi:10.3389/fneur.2018.00861)

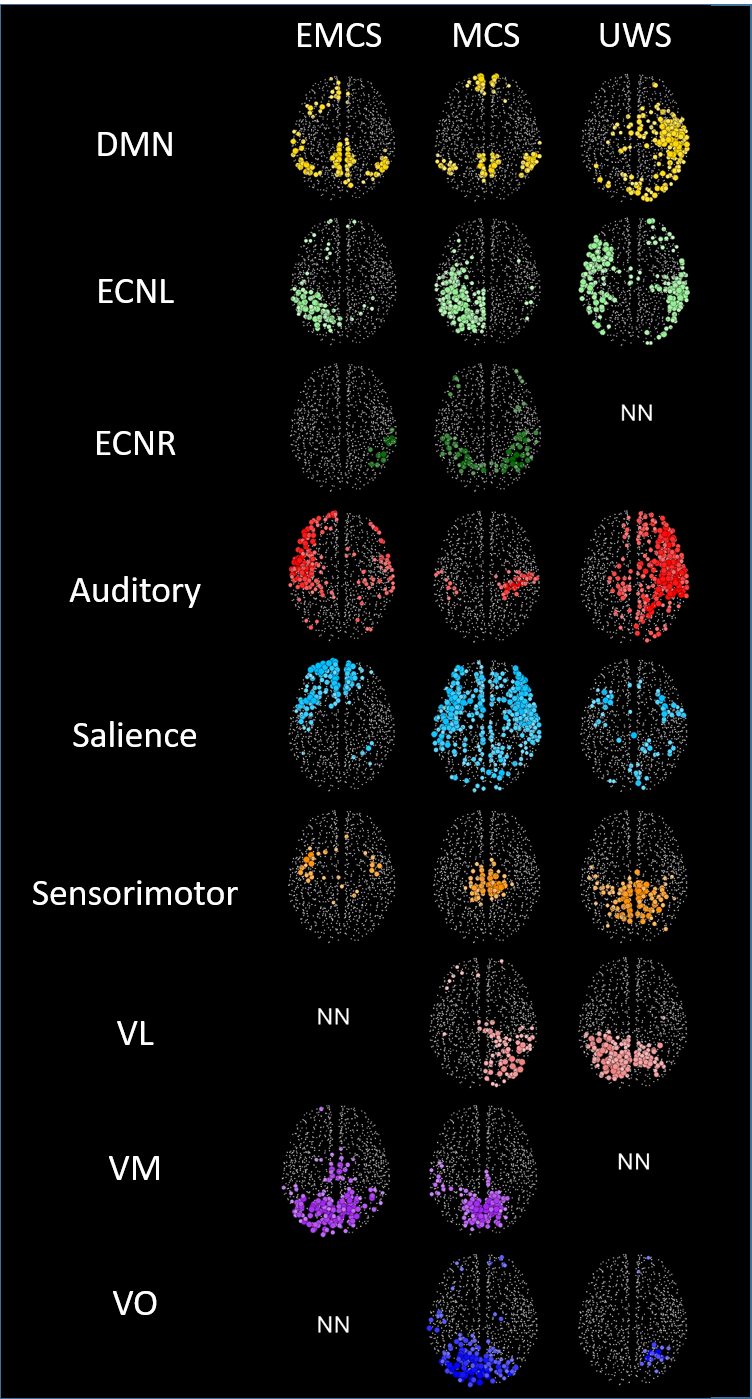

Supplement: Figure S1 — GS scalar maps of the nine RSNs of patients in EMCS, MCS, and UWS. From the two acquisitions, only the networks classified as neuronal are shown. When both acquisitions had neuronal components, the highest ROF value was used to choose the best spatial pattern of the network. The size of the circle represents the strength of the GS. The darker the circle, the higher the GS. Only the GS values greater than 0.5 of the maximum GS value of that network are plotted. [file Image_1.TIFF]

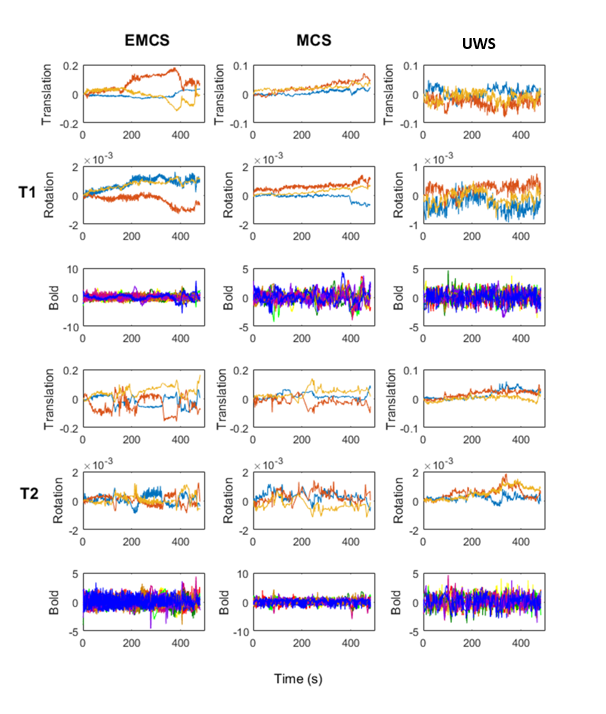

Supplement: Figure S2 — Motion curves illustrate translation (in mm) for x (blue), y (red), and z (orange) and rotation (in °) for pitch (blue), roll (red), and yaw (orange) parameters, and the time courses of each the nine RSNs (auditory, DMN, ECNL, ECNR, salience, sensorimotor, VL, VM, and VO) over 480 s. [file Image_2.TIFF]

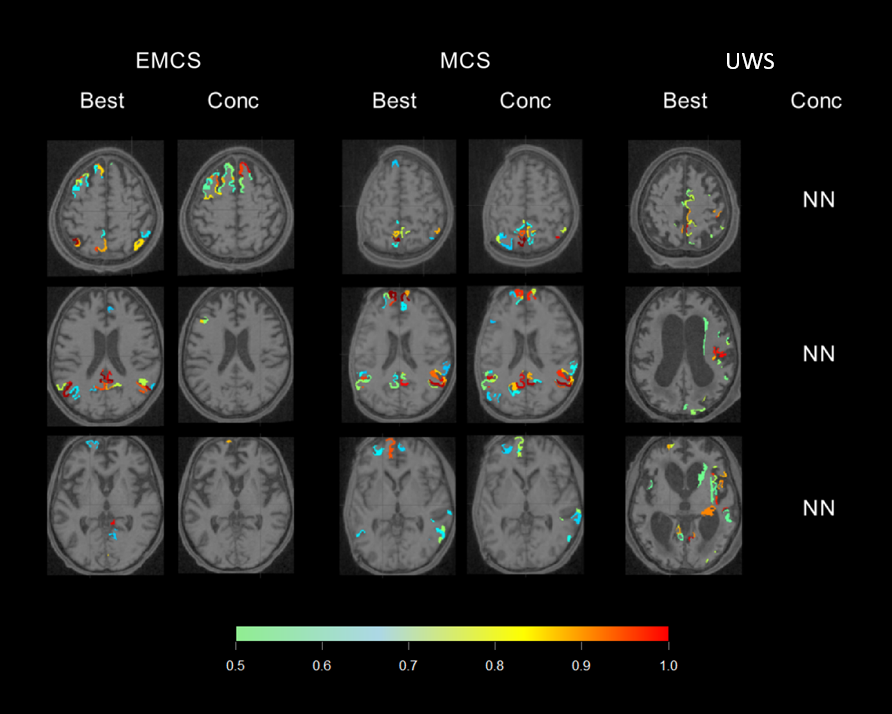

Supplement: Figure S3 — Three most representative axial slices of the GS implemented on the normalized structure of the DMN network are presented for the three patients for the best functional pattern and concatenated data. [file Image_3.TIFF]

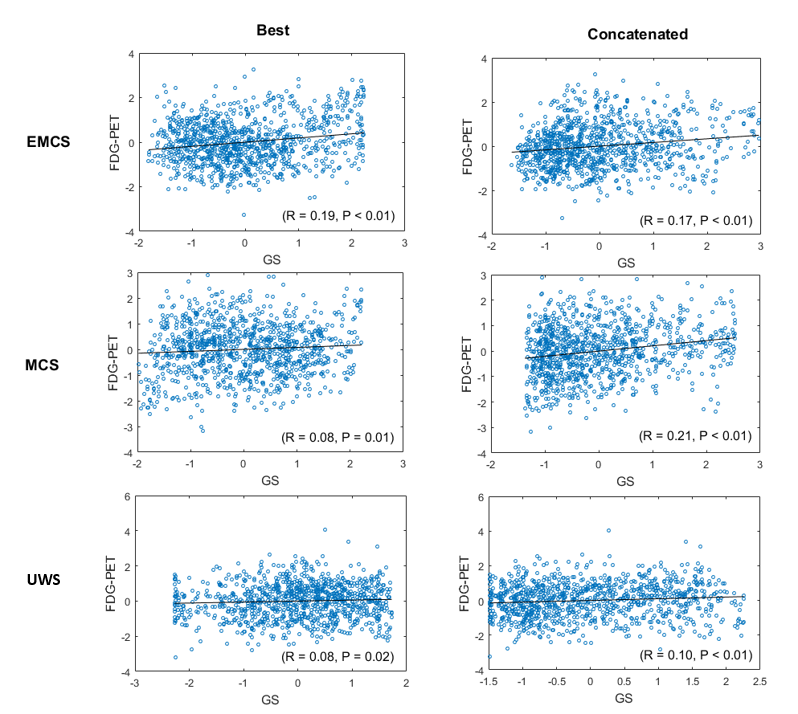

Supplement: Figure S4 — Scatter plots for the EMCS, MCS and patients in UWS showing the correlation between the FDG-PET and GS of the 1015 parcellated ROI. Solid line indicates the best linear fit to the data and on the northeast corner of each scatter plot the linear correlation value is reported along with its statistical p-value. [file Image_4.TIFF]

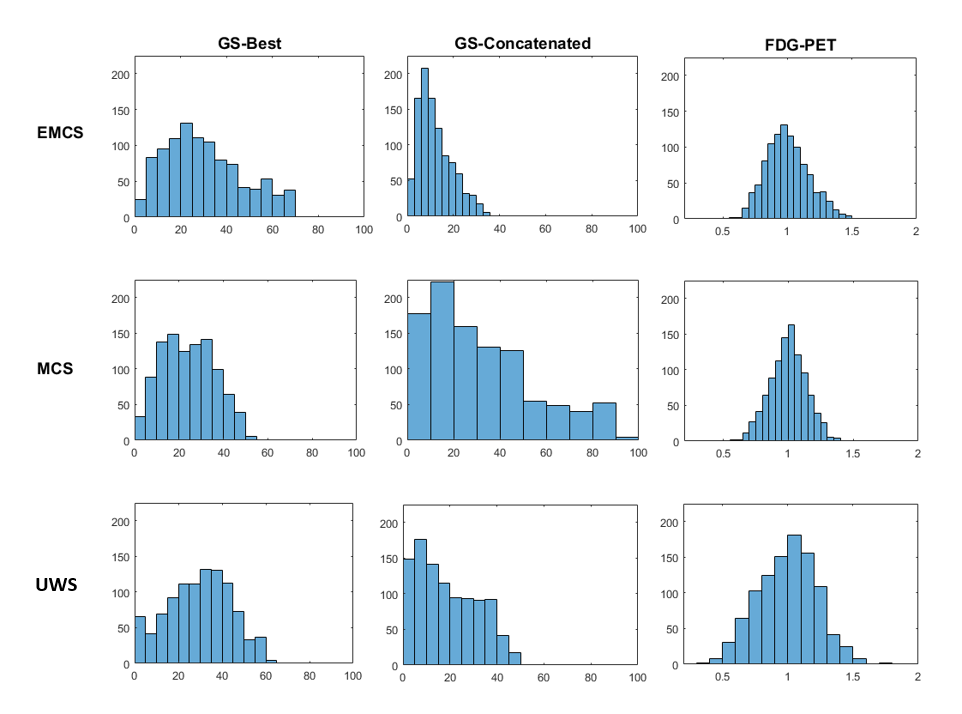

Supplement: Figure S5 — Distribution plots of GS for the best acquisition, concatenated data and FDG-PET for patients in EMCS, MCS and UWS. [file Image_5.TIFF]
