# Supplementary material for: Multimodal Neuroimaging Approach to Variability of Functional Connectivity in Disorders of Consciousness: A PET/MRI Pilot Study
Source: Front Neurol. 2018 Oct 18;9:861. doi: 10.3389/fneur.2018.00861 (PMC6200912; doi:10.3389/fneur.2018.00861)
Supplement: Table S1 — Coma Recovery Scale-Revised total and subscores in the three patients collected in the day of neuroimaging and 5 days in 1 week before and after. [file Table_1.docx]

Table S1. Coma Recovery Scale-Revised total and subscores in the three patients collected in the day of neuroimaging and five days in one week before and after.

| Assessment | Case 1  UWS | Case 2  MCS | Case 3  EMCS |
| --- | --- | --- | --- |
| 1 | 6 (2-0-1-2-0-1) | 11 (3-3-2-1-0-2) | 22 (3-5-6-3-2-3) |
| 2 | 6 (2-0-1-2-0-1) | 11 (3-3-2-1-0-2) | 21 (3-5-5-3-2-3) |
| 3 | 6 (2-0-1-2-0-1) | 10 (3-3-2-0-0-2) | 21 (3-5-5-3-2-3) |
| 4 | 6 (1-0-2-2-0-1) | 11 (3-3-2-1-0-2) | 20 (3-5-5-2-2-3) |
| 5 | 6 (1-0-2-1-0-2) | 11 (3-3-2-1-0-2) | 21 (3-5-5-3-2-3) |
| *6 | 6 (1-0-2-1-0-2) | 11 (3-3-2-1-0-2) | 22 (4-5-5-3-2-3) |
| 7 | 6 (1-0-2-1-0-2) | 11 (3-3-2-1-0-2) | 22 (4-5-5-3-2-3) |
| 8 | 6 (2-0-1-1-0-2) | 10 (3-3-2-0-0-2) | 21 (3-5-5-3-2-3) |
| 9 | 6 (2-0-1-1-0-2) | 10 (3-3-2-1-0-1) | 20 (3-5-5-2-2-3) |
| 10 | 6 (2-0-1-1-0-2) | 11 (3-3-2-1-0-2) | 20 (3-5-5-2-2-3) |
| 11 | 6 (2-0-1-1-0-2) | 11 (3-3-2-1-0-2) | 21 (4-5-5-2-2-3) |

Note. CRS-R=coma recovery scale-revised; UWS= unresponsive wakefulness syndrome; MCS=minimally conscious state; EMCS=emergence from MCS.

* indicates neuroimaging day. The changes in CRS-R total or subscores are marked in bold
