# Supplementary material for: Multimodal Neuroimaging Approach to Variability of Functional Connectivity in Disorders of Consciousness: A PET/MRI Pilot Study
Source: Front Neurol. 2018 Oct 18;9:861. doi: 10.3389/fneur.2018.00861 (PMC6200912; doi:10.3389/fneur.2018.00861)
Supplement: Appendix 1 — Classification criteria for visual analysis of EEG background activity. [file Data_Sheet_1.docx]

**Appendix 1.** Classification criteria for visual analysis of EEG background activity

| **EEG background activity category** | **EEG features** |
| --- | --- |
| Normal | Presence of predominant posterior alpha rhythm and of the APG, without focal or hemispheric slowing or epileptiform abnormalities |
| Mildly abnormal | Presence of predominant posterior theta activity (≥20 μV), symmetric or not, with frequent (10-49% of recording) posterior alpha rhythms |
| Moderately abnormal | Presence of predominant posterior theta activity (≥20 μV), symmetric or not, poorly organized APG, even with rare (<1% of recording) or occasional (1-9% of recording) posterior alpha rhythms |
| Diffuse slowing | Presence of predominant diffuse theta or theta/delta rhythms at amplitude ≥20 μV, without APG |
| Low voltage | Presence of predominant EEG activity (theta or delta) <20 μV over most brain regions |

**Clinical sketches of patients**

**Case 1 (patient in UWS)**. A 43-year-old woman was admitted to the intensive care unit because of cardio-respiratory arrest following septic shock with rhabdomyolysis in October 2016. After the severe anoxic brain injury, she remained in a comatose state for 20 days and then recovered arousal with cyclic eye opening. When she was admitted to the neurorehabilitation unit, 50 days after injury, the patient was classified to be in UWS by repeated behavioural assessments using Coma Recovery Scale-Revised (CRS-R). The diagnosis was confirmed by serial assessments during the following 6 months, on the day of MR/PET session and in the weeks before and after MR/PET session. Across repeated evaluations, CRS-R evidenced presence of sound localization (auditory scale= 2), abnormal posture in response to nociceptive stimulus (motor scale= 1), reflexive chewing movements following introduction of tongue blade (oro-motor scale= 1) and eye opening upon stimulation (arousal scale= 1), whereas no behavioural responses were elicited by visual stimuli or communication was present (visual and communication scales= 0). Some inconsistent behavioural changes were only observed on auditory and motor scales: on some evaluations, the patients showed a clear startle response to auditory stimuli but no localization to sound, whereas her right upper limb sometimes moved away from the point of nociceptive stimulation. Nonetheless, her CRS-R total score did not change in the weeks before and after MR/PET session.

**Case 2 (patient in MCS).** A 18-year-old boy was hospitalized in a comatose state because of a severe closed brain injury due to a car accident in September 2017. He underwent a left temporo-parietal decompressive craniectomy and emptying of a wide left epidural hematoma. Twenty days later he was admitted to our neurorehabilitaton unit and diagnosed to be in UWS/VS. In one month he recovered visual tracking and reproducible movements to command such as eyes closure, head and left arm movements (CRS-R total score=11). He was enrolled in the study 3 months after brain injury, when he was in stabilized medical conditions. At that time his diagnosis was MCS and the patients showed reproducible eye, head and arm movements to verbal command (auditory scale= 3), visual pursuit (visual scale=3), arm flexion withdrawal to nociceptive stimulus (motor scale= 2), reflexive chewing movements following introduction of tongue blade (oro-motor scale= 1) and eye opening without stimulation (arousal scale= 2), whereas communication was not present (communication scales= 0). Repeated clinical assessments allowed to notice inconsistent oral reflexive movements in the days before (3^rd^ day) and after the RM/PET session (8^th^ and 9^th^ day), thus CRS-R total score ranged 10-11 without change in clinical diagnosis.

**Case 3 (patient in EMCS).** A 57-year-old man acutely developed a severe anoxic brain injury following a cardiac arrest after his first heroin dose in March 2017. Fifteen days later, he was admitted to our neurorehabilitation unit in clinical condition of UWS. About 2 months after brain injury, he recovered ability to follow verbal commands such as “open your mouth and close your eyes” (auditory scale= 3) and automatic motor responses (motor scale=5) and then, 2 months later, the ability to pursuit a moving object or person (visual scale=3), to recognize two distinct objects (visual scale= 5) and to follow object-related verbal commands, such as “look at the glasses” (CRS-R auditory scale=3). At 5 months post injury, he further recovered functional and appropriate verbal communication, thus emerging from MCS (CRS-R total score= 21). During the subsequent 25 days, his serial CRS-R total scores ranged 20-22, since the patient did not show consistent movement to command or automatic motor responses in all CRS-R assessments. Because of agitation, low-dosage quetiapine was administerd; we stopped quetiapine 15 hours before PET/MRI scanning, as foreseen by our study protocol. On the day of neuroimaging acquisition, his CRS-R total score was 22.
